# Discrete Scale Invariance of Human Large EEG Voltage Deflections is More Prominent in Waking than Sleep Stage 2

**DOI:** 10.3389/fnhum.2015.00638

**Published:** 2015-12-02

**Authors:** Todd Zorick, Mark A. Mandelkern

**Affiliations:** ^1^Greater Los Angeles Veterans Administration Healthcare System, Departments of Psychiatry, (TZ) and Imaging (MM), University of CaliforniaLos Angeles, CA, USA; ^2^Department of Psychiatry and Biobehavioral Sciences, University of CaliforniaLos Angeles, CA, USA; ^3^Department of Physics, University of CaliforniaIrvine, CA, USA

**Keywords:** consciousness, neuronal avalanche, scale invariance, discrete scale invariance, EEG, human, sleep, electroencephalography

## Abstract

Electroencephalography (EEG) is typically viewed through the lens of spectral analysis. Recently, multiple lines of evidence have demonstrated that the underlying neuronal dynamics are characterized by scale-free avalanches. These results suggest that techniques from statistical physics may be used to analyze EEG signals. We utilized a publicly available database of fourteen subjects with waking and sleep stage 2 EEG tracings per subject, and observe that power-law dynamics of critical-state neuronal avalanches are not sufficient to fully describe essential features of EEG signals. We hypothesized that this could reflect the phenomenon of discrete scale invariance (DSI) in EEG large voltage deflections (LVDs) as being more prominent in waking consciousness. We isolated LVDs, and analyzed logarithmically transformed LVD size probability density functions (PDF) to assess for DSI. We find evidence of increased DSI in waking, as opposed to sleep stage 2 consciousness. We also show that the signatures of DSI are specific for EEG LVDs, and not a general feature of fractal simulations with similar statistical properties to EEG. Removing only LVDs from waking EEG produces a reduction in power in the alpha and beta frequency bands. These findings may represent a new insight into the understanding of the cortical dynamics underlying consciousness.

## Introduction

Although rarely explicitly stated, the dominant model for the analysis of human EEG signals for more than 50 years has been spectral analysis, implicitly viewing the time-dependent changes in cortical local field potentials as a set of dynamic and standing electrical waves originating from the top 5 mm of the cerebral cortex (Nunez and Srinivasan, 2006; Nunez, 2010). Such techniques have proven spectacularly successful in both research and clinical medicine (Schwilden, 2006; Arciniegas, 2011; Schiff et al., 2014).

However, many lines of evidence originating in multielectrode array recordings of rodent cortex (Plenz and Thiagarajan, 2007; Gireesh and Plenz, 2008; Klaus et al., 2011), extending through human electrocorticography (Priesemann et al., 2013), electroencephalography (EEG; Poil et al., 2012; Palva et al., 2013), magnetoencephalography (MEG; Shriki et al., 2013; Yu et al., 2013), and magnetic resonance imaging (MRI; Kitzbichler et al., 2009) have resounding demonstrated that a fundamental organizing principle of neuronal activity in the cerebral cortex is via dynamic distributions of scale-free “neuronal avalanches”. Power-law-distributed neuronal avalanches have been shown to demonstrate many hallmarks of dynamic systems in a critical state (Shriki et al., 2013; Yu et al., 2013), which simulations have shown are likely to lead maximal information transmission throughout the neuronal ensemble (reviewed in Shew and Plenz, 2013). *A priori*, such non-stationary dynamical systems would not be best analyzed via spectral analysis (Weisstein, 2014).

EEG has also been linked to neuronal avalanche activity. The developmental time course of neuronal avalanche maturation was studied in a rodent model, where the onset of power-law distributed neuronal avalanches was correlated with the maturation of beta and gamma band EEG signals (Gireesh and Plenz, 2008). Neural network models with avalanche dynamics can quite readily produce a signal that approximates human EEG, however with reduced power in the 20–50 Hz (“beta” and “gamma” bands; de Arcangelis and Herrmann, 2012). Many convincing demonstrations of neuronal avalanche activity in rodents, non-human primates, and humans have been reported, showing that functional mammalian cortical neuronal ensembles organize into scale-free, critical-state dynamics, with a characteristic power law exponent and branch ratio (Shew and Plenz, 2013; Shriki et al., 2013). Additionally, EEG and MEG signals themselves have been shown to be highly non-stationary, albeit amenable to analysis as “quasi-stationary” (Kaplan et al., 2005).

While compelling, these observations beg the question as to what role, if any, neuronal avalanches may play in the origin of conscious mental states, especially given the clear observations that rodent brain tissue *in vitro* exhibits similar power law behavior and critical state dynamics via branch ratio analysis of avalanches to that of waking human MEG signals (Plenz and Thiagarajan, 2007; Shriki et al., 2013). In fact, cortical neuronal avalanche size has been found to follow a power law with an exponent of −1.5 in rodent cortex cultures *in vitro*, *in vivo* anesthetized rodent cortex, and awake primates (Klaus et al., 2011). Consciousness is thought to be mediated at the neuronal level via cortical-thalamic feedback loops capable of global informational binding without strict localization (Edelman and Tonioni, 2000; Baars et al., 2013; Boly et al., 2013), and many investigators have pointed out the theoretical advantages of critical-state neuronal dynamics in terms of rapid, global cortical information transfer (Poil et al., 2012; Palva et al., 2013; Shew and Plenz, 2013). Cortical avalanche dynamics have been explored using power law techniques in awake vs. sleeping rodents, and found to have a very similar structure (Ribeiro et al., 2010). By contrast, avalanche distributions were found to differ somewhat among sleep stages in a group of human epileptic patients implanted with hippocampal electrodes (Priesemann et al., 2013).

Investigators have also studied changes in neuronal physiology with conscious vs. unconscious mental states using methods looking for Long Range Temporal Correlation (LRTC) in neuronal physiology in many different systems (Li et al., 2008; Nikulin et al., 2012; Blythe et al., 2014). Using multiscale entropy and detrended fluctuation analysis (DFA) of renal sympathetic nerve activity, anesthetized rats were found to have decreased LRTC compared to awake rats (Li et al., 2008). Using Hilbert transformed alpha and gamma band information, subsequent application of DFA demonstrated that subjects with schizophrenia had decreased alpha and beta band LRTC compared to healthy controls (Nikulin et al., 2012). A further application of LRTC in EEG demonstrated that an improved source localization method was able to improve LRTC estimates using DFA (Blythe et al., 2014).

With an eye to these data, we make the fairly obvious observation that critical-state neuronal avalanche dynamics are unable to explain much of the regularity in structure seen in waking human EEG signals, both when analyzed by spectral analysis and autocorrelation function (ACF) analysis. Spectral analysis and ACF analysis yield markedly diferent results for waking and sleep 2, whereas cortical avalanche dynamics have been shown to be very similar across different states of cortical function, both *in vivo* and *in vitro* (Plenz and Thiagarajan, 2007; Klaus et al., 2011; Shriki et al., 2013). We utilized a publicly available EEG dataset, taking advantage of continuous somnogram information to get waking and sleep stage EEG tracings from the same individuals, in order to directly compare changes in EEG large voltage deflection (LVD) dynamics within subjects. Taking the viewpoint that ensembles of neuronal avalanches contribute to EEG, we hypothesized that discrete scale invariance (DSI) might be superimposed on critical-state dynamics in waking more than in sleep stage 2 LVDs. This is because log-periodic oscillations in the observables of natural processes can be caused by a partial breaking of scale invariance, resulting in a “periodicity” in the data (cf. Sornette, 1998, 2006; Zhou et al., 2005). This hypothesis could represent a new insight into our understanding of the cortical neuronal dynamics of brain function, including consciousness.

For the following brief description of DSI, we adapt from several expositions by Sornette (1998, 2006). This report is too brief to provide a comprehensive review of DSI, for which we refer the interested reader to Sornette (1998) and references therein. Power-law-distributed systems are typically characterized by continuous scale invariance, where an observable function *O(x)* is scale invariant under the arbitrary change *x* → λ*x* (where *λ* → 1^+^) when there is a function *μ*(λ) such that:

(1) O(x)=μO(λx)

The solution to which is the basic power law formula:

(2) $$O(x)=Cx^{a}$$

where *C* is a constant and

(3) $$\alpha =-\frac{\log\mu }{\log\text{λ}}$$

Studies in complex systems prone to catastrophes, including financial markets, ruptures of pressurized containers, and earthquakes have demonstrated that certain dynamical systems can exhibit a weaker form of DSI where the control parameter *λ* acts as a scaling ratio, and can only exhibit discrete values, e.g., *λ*_1_,*λ*_2_, *λ*_3_…, *λ*_n_ (Sornette, 1998, 2006). As a result, in DSI, the power law behavior of the underlying system is also controlled by complex exponents decorating the power laws, leading to periodicity (Sornette, 1998, 2006):

(4) $$\alpha =-\frac{\log\mu }{\log\text{λ}}+i\frac{2\pi n}{\log\text{λ}}$$

where *n* is an integer and *i* the imaginary unit. In the case of *n* = 0, the imaginary part drops out and continuous scale invariance is recovered as in (3).

## Materials and Methods

### EEG Data

We utilized a publicly available database of single-channel EEG recordings from the MIT-BIH polysomnographic database recorded using the 10–20 EEG reference system (slpdb; http://www.physionet.org; Ichimaru and Moody, 1999; Goldberger et al., 2000). We utilized the same dataset previously reported, and details of the subjects and EEG data utilized are as described (Zorick and Mandelkern, 2013). Briefly, our database consists of eight different non-contiguous 1 min EEG tracings (recorded at 250 Hz) from 14 subjects in both waking and sleep stage 2 consciousness, one lead per subject. Approval for this study was provided by the local VA West Los Angeles Institutional Review Board.

### Simulations

Our choice of simulations was motivated by the fact that these simulations exhibit similar fractal (and in some cases, multifractal) properties to that of human waking EEG (Zorick and Mandelkern, 2013). The log normal sigma 0.1 multifractal series (32,768 data points) was downloaded from http://www.physionet.org/physiotools/multifractal/, made from the log-normal wavelet cascade algorithm with parameters *v* = *ln(2)/4* and *σ = 0.1* as described (Muzy et al., 1993). The fractional Brownian motion monofractal series was generated with Hurst exponent (H) of 0.2 using the “dvfBm” R package (120,000 data points; version 1.0; Coeurjolly, 2001). The binomial multifractal series (BMS) was constructed with 120,000 data points after Kantelhardt et al. (2002); Zorick and Mandelkern (2013). The BMS is a series of *N* = ${}^{2^{n_{max}}}$ numbers with index *k* = 1, …, N, defined by

(5) $$x_{k}=a^{n(k-1)}{\left(1-a\right)}^{n_{\max}-n(k-1)}$$

For this series, *a* is a user-defined parameter which can take values 0.5 < *a* < 1. We chose the parameter *a* = 0.6 such that the resulting multifractal spectrum roughly matches that of the human waking EEG samples (Zorick and Mandelkern, 2013). Here *n(k)* is the sum of digits equal to 1 in the binary representation of the index *k* (120,000 data points). As an example, choosing an index value of *k* = 13, *n*_(13)_ = 3, as the binary representation of the decimal number 13 is 1101.

### Spectral Analysis and Autocorrelation Function

Fast Fourier Transform (FFT) and ACF analysis were performed on EEG tracings utilizing the R “spec.pgram” and “acf” functions (RCoreTeam, 2012).

### EEG Large Voltage Deflections

Based upon data from MEG showing that only spikes >3 *SD* from the mean in size are able to be differentiated from the Gaussian distribution (Shriki et al., 2013), we identified LVDs of size >2.5 *SD* from the mean of the EEG segment, separately both for positive and negative voltages. The results obtained are quite similar when identifying LVDs of size >2 *SD* from the mean. However, the relatively short length of available EEG traces and the relatively coarse sampling rate (250 Hz) limited analysis in our dataset to thresholds <3 *SD* from the mean, as thresholds higher than this would not generate enough LVDs to reliably analyze in subsequent steps. We defined LVD size s as the sum of voltage readings between times at which the EEG voltage is zero (zero crossings). We utilized LVD size as an appropriate measure for further analysis, as it led to robust PDF estimations given adequate sequence length. Each EEG segment was analyzed separately for LVD PDF estimation and periodicity.

### Probability Density Function Analysis

We utilized the R “density” function (RCoreTeam, 2012) to obtain the normalized probability density function (PDF) *f(s)* for each LVD segment. Segments with fewer than 50 avalanches per segment did not produce reliable PDF estimates, so these were excluded. After PDF estimation, the scale sizes *f(s)* were natural log transformed for further analysis.

### Lomb-Scargle Periodogram Analysis

To assess for DSI in EEG LVD sizes, we utilized the Lomb-Scargle nonparametric periodogram method from the log-scale PDF *f(s)* of the neuronal avalanches. The Lomb-Scargle method is motivated as the necessary logarithmic transformation step of the frequencies presents non-uniformly spaced samples (Zhou and Sornette, 2002; Zhou et al., 2005; Press et al., 2007). The R function “spec.ls” in the “cts” package (written by Zhu Wang) was used, which was designed to follow (Press et al., 2007). Normalized Lomb-Scargle periodograms were constructed by dividing by the variance of the frequency data *f(s)* to permit statistical testing (Press et al., 2007). We utilized normalized Lomb-Scargle periodograms to assess for statistical significance using the probability threshold of *p* = 0.01, with *P(>z)* ≈ *Me*^-z^, where *z* is the threshold value at the desired significance level, and *M* is a maximum estimate of the number of independent frequencies in the periodogram (which we heuristically estimate at twice the number of data points in the input series (512 data points; Press et al., 2007).

### Statistical Analysis

All statistical analyses were performed using R (RCoreTeam, 2012) and SPSS (IBM, Chicago, 2014). Statistically significant Lomb-Scargle periodogram peaks were broadly defined as any point with a positive slope immediately preceding, and a negative slope immediately following. For each segment we have the number of significant (*p* < 0.01) Lomb-Scargle peaks and the area of the Lomb-Scargle periodogram above the *p* = 0.01 probability threshold, for both positive- and negative-LVD periodograms. We analyze these four data sets separately using a linear mixed model, regressing the number of peaks (or area) against consciousness condition (waking or sleep stage 2). The covariance model for the within-subject measurements is taken as compound symmetry.

## Results

### Waking EEG Differs from Sleep Stage 2 EEG by Both Spectral Analysis and Autocorrelation Function Analysis

**Figure 1:**
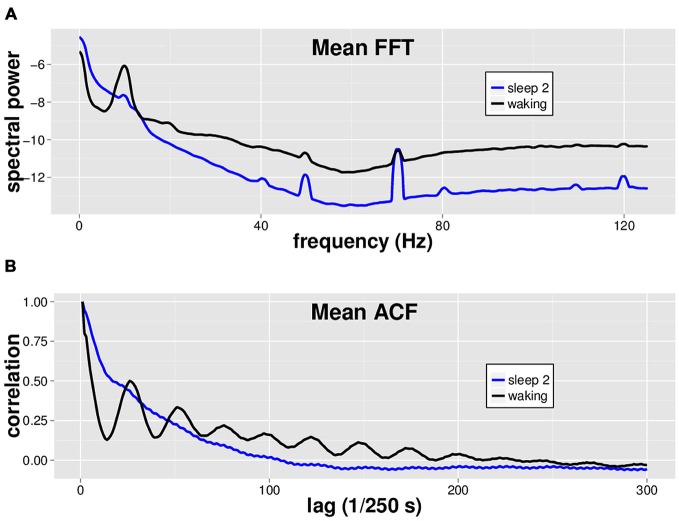
**Mean spectral analysis and autocorrelation function analysis for waking vs. sleep stage 2 EEG.** Fourteen subjects had 8 min each of both waking and sleep stage 2 EEG data, in non-continuous 1 min segments. **(A)** Fast Fourier Transform (FFT) was performed on 8 × 1 min segments of both waking (black) and sleep stage 2 (blue) EEG for each subject, and the spectra were averaged across segments and subjects to generate the mean spectrum shown. Waking EEG has more power in the alpha (8–13 Hz), beta, and gamma band (~20–40 Hz) ranges. **(B)** Autocorrelation function (ACF) with a maximum lag of 500 data points was performed on 8 × 1 min EEG segments of both waking (black) and sleep stage 2 (blue) EEG for each subject. Note characteristic oscillatory pattern for waking EEG ACF.

Using a dataset with 8 min of EEG from both waking and sleep stage 2 in 14 individuals, we performed spectral analysis via FFT and ACF analysis on each of eight separate non-contiguous 1 min tracings for each subject (Figure 1). While by no means a novel finding, we simply make the observation that waking EEG is readily distinguished from sleep stage 2 EEG via spectral analysis (Figure 1A). We furthermore note the characteristic oscillatory patterns in the ACF that differ markedly between waking and sleep stage 2 EEGs (Figure 1B).

### LVD PDFs Appear to have a Similar Structure for Waking and Sleep Stage 2, though more Avalanches are found in Waking

**Figure 2:**
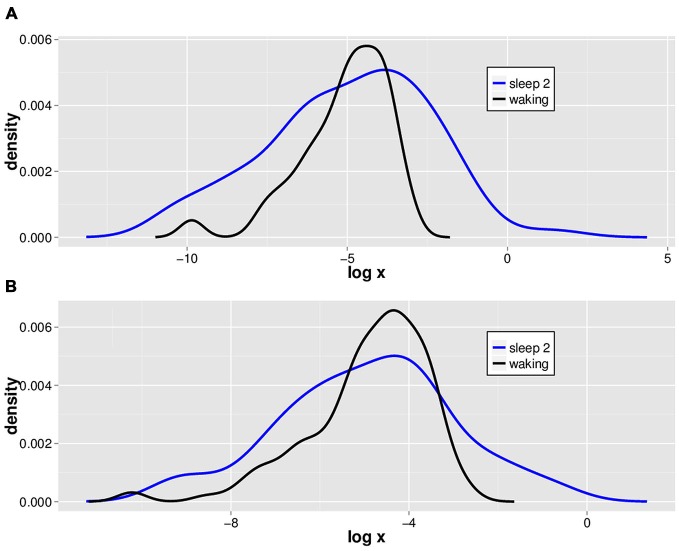
**Typical natural log scale probability distribution estimations for positive and negative LVDs.** LVDs were isolated at 2.5 *SD* from positive **(A)** and negative **(B)** voltage deflections from a 1 min segment of waking (black) and sleep stage 2 (blue) EEG tracings from one subject for illustration purposes.

We isolated 2.5 *SD* LVDs from the 14 subjects’ waking and sleep stage 2 EEGs, and show a semilog plot of the PDF vs. LVD size for one subject (Figure 2). Positive (Figure 2A) and negative (Figure 2B) LVD PDFs appear somewhat similar for both waking and sleep stage 2.

By contrast, we observe a clear distinction between waking and sleep stage 2 in terms of both the mean number of LVDs per segment, and the percentage of segments containing more than 50 LVDs, both shown by statistical comparison to be highly significant (Table 1). The number of segments we were able to utilize per state of consciousness for both positive and negative LVDs is listed in Table 1.

**Table 1:** **Waking EEGs have more LVDs than sleep 2**.

A	^#^LVDs per segment	Statistic^1^	*p*-value
**Pos**
Waking	141.5 (55.4)	*t*_(111)_ = 10.75	<0.001
Sleep 2	70.6 (56.7)
**Neg**
Waking	159.5 (82.4)	t_(111)_ = 11.34	<0.001
Sleep 2	60.7 (44.0)

**B**	**^#^Segments with >50 LVDs**	**Statistic^2^**	*p-value*

**Pos**
Waking	109 (97%)	χ^2^ = 48.2	<0.001
Sleep 2	57 (51%)
**Neg**
Waking	106 (95%)	χ^2^ = 55.1	<0.001
Sleep 2	47 (42%)

^1^*Pairwise t-test; ^2^McNemar’s chi-square test; A: numbers are listed as Mean (SD) of # of LVDs per segment; B: numbers are listed as N (%) out of 112 total segments*.

### Waking Differs from Sleep Stage 2 in the Extent of DSI Seen in the EEG LVD PDF Fluctuations

**Figure 3:**
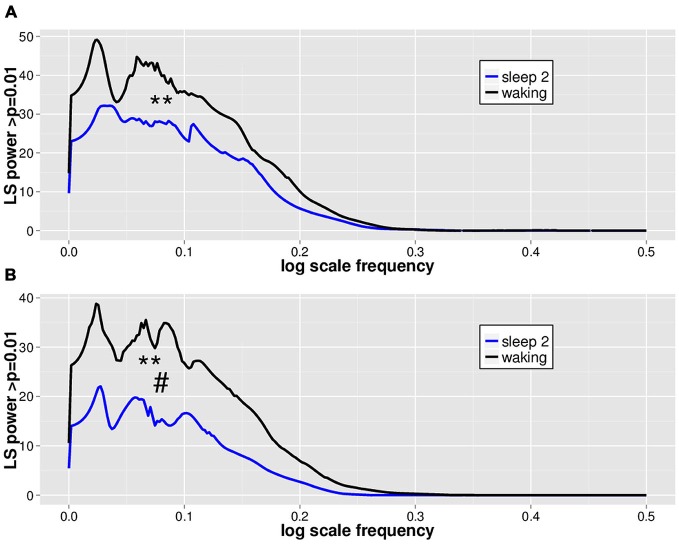
**Mean significant Lomb-Scargle normalized periodogram areas from PDF plots of log scale frequencies for positive (A) and negative (B) LVDs.** Data represent mean normalized Lomb-Scargle periodogram plots of power > *p* = 0.01 across 14 subjects for 8 min of EEG for waking (black) and sleep stage 2 (blue) LVDs. **p < 0.001 for mean number of significant peaks for waking vs. sleep 2 by linear mixed effect modeling. ^#^*p* < 0.05 for total significant area for waking vs. sleep 2 by linear mixed effect modeling; see Table 2.

LVD PDFs were subjected to log transformation for Lomb-Scargle periodogram analysis (Figure 3) to look for evidence of DSI in the PDF as a function of LVD size. While the total mean area of statistically significant periodogram signals does not differ between waking and sleep 2 positive LVDs, there is a modest difference in total mean area for negative LVDs (Figure 3; Table 2). However, for both positive and negative LVDs, there were clearly more statistically significant periodogram peaks for waking compared to sleep stage 2 (Figure 3; Table 2).

**Table 2:** **LS periodogram from PDF statistics**.

Pos	Mean (*SD*)	*F_(1,151)_*	*p*-value
**Total area**
Waking	3443 (5206)	1.61	0.21
Sleep 2	2379 (5295)
**Peaks**
Waking	3.91 (3.44)	18.25	<0.0001
Sleep 2	1.82 (2.29)

Neg	Mean (*SD*)	*F*_(1, 138)_	*p*-value

**Total area**
Waking	2647 (4122)	3.94	0.049
Sleep 2	1306 (2768)
**Peaks**
Waking	3.87 (3.61)	17	0.0001
Sleep 2	1.91 (2.43)

*Pos, positive LVDs; Neg, negative LVDs. Statistics listed are derived from linear mixed effect modeling*.

As a further test of this hypothesis, in order to confirm that this effect persisted for longer segment lengths, we isolated additional 5 min long continuous EEG tracings from the same subjects for both waking and sleep stage 2 (all 14 subjects, one 5 min segment for each state of consciousness). These were then processed using the same techniques of isolation of 2.5 *SD* LVDs, followed by PDF estimation, log transformation, and Lomb-Scargle periodogram analysis (Table 3). Given that there is less data per subject in this analysis, it is unsurprising that there is now only a strong trend to significance for positive LVD areas (*p* = 0.11) and peaks (*p* = 0.06), with no difference for negative LVD areas (*p* = 0.28) and peaks (*p* = 0.25; Table 3). However, in all cases the average values for waking EEGs are greater than those for sleep 2 EEGs, supporting the patterns seen for the 1 min EEG tracing analysis (Tables 2, 3; Figure 3).

**Table 3:** **LS periodogram from PDF statistics of 5 min long EEG segments**.

Pos	Mean (*SD*)	*F*_(1,151)_	*p*-value
**Total area**
Waking	521.3 (1194.9)	*V* = 14^a^	0.11
Sleep 2	26.7 (94.3)
**Peaks**
Waking	1.21 (2.04)	*t* = 2.02^b^	0.06
Sleep 2	0.14 (0.36)

**Neg**	Mean (*SD*)	**Statistic**	*p*-value

**Total area**
Waking	527.8 (1327.7)	*V* = 12^a^	0.28
Sleep 2	16.3 (41.5)
**Peaks**
Waking	0.86 (1.75)	*t* = 1.2^b^	0.25
Sleep 2	0.29 (0.61)

*Pos, positive LVDs; Neg, negative LVDs. ^a^Wilcoxon Signed Rank test statistic; ^b^Paired t-test statistic*.

### Simulation Data and LVD DSI

**Figure 4:**
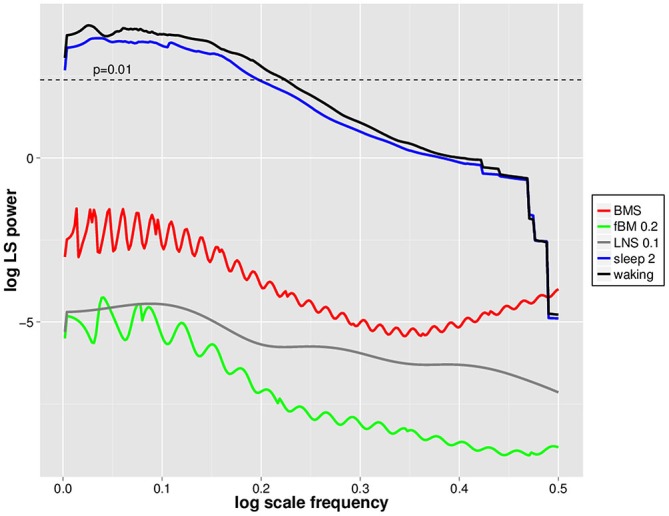
**Natural log scale normalized Lomb-Scargle periodgrams for waking, sleep stage 2, log normal sigma 0.1 (LNS 0.1), binomial multifractal series (BMS; a = 0.6), and fractional Brownian Motion (fBM 0.2) avalanches.** Data represent mean natural log of normalized Lomb-Scargle periodogram plots of 2.5 *SD* positive LVDs across 14 subjects for 8 min EEG from waking (black) and sleep stage 2 (blue), along with the same measures for a single LNS 0.1 multifractal series (gray), BMS (red), and fBM 0.2 (green). Horizontal dashed line indicates the threshold for statistical significance of normalized Lomb-Scargle periodogram peaks (*p* = 0.01) based upon the number of possible frequencies in the PDF estimation, as defined in Press et al. (2007).

In order to evaluate whether DSI is specific to waking EEG avalanches, we utilized mono- and multifractal simulation data which have similar fractal dynamics (as assessed by Hölder exponents) to waking EEGs (cf. Zorick and Mandelkern, 2013). Positive 2.5 *SD* amplitude deflections were isolated from BMS, fractional Brownian Motion with H = 0.2 (fBM 0.2) and log normal sigma 0.1 (LNS 0.1) multifractal series, and subjected to PDF estimation and log transformation followed by normalized Lomb-Scargle periodogram analysis as for EEGs (Figure 4). For perspective, these were plotted together with mean waking and sleep stage 2 positive LVD normalized Lomb-Scargle periodogram data derived from a natural log scale (Figure 4). Significance level for normalized Lomb-Scargle periodogram peaks are shown with a horizontal line for the *p* = 0.01 significance level (Figure 4). As can be seen, normalized Lomb-Scargle periodogram data of 2.5 *SD* positive deflections from BMS, LNS 0.1, and fBM 0.2 do not approach significance, whereas the mean waking and sleep stage 2 LVDs clearly exceed the significance threshold (Figure 4). This indicates that DSI is not a general feature of fractal series with mean Hölder exponents of ~0.2, but rather specific for EEG-derived LVDs (Figure 4).

### Removal of EEG LVDs Reduces Alpha and Beta Spectral Power

**Figure 5:**
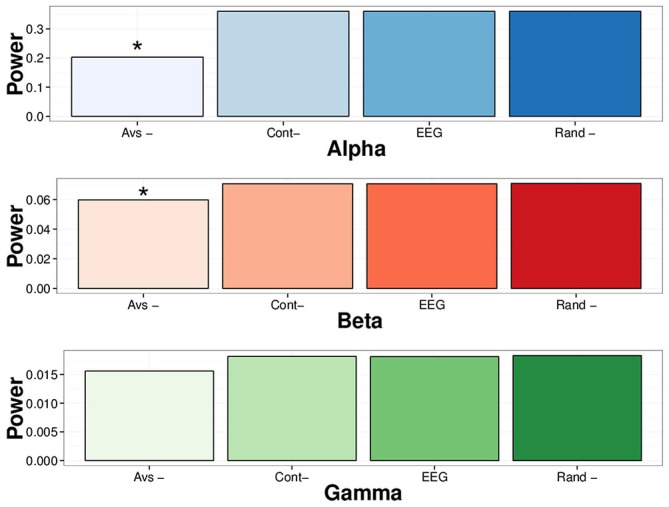
**Removing all 2.5 *SD* LVDs (positive and negative) reduces spectral power in alpha and beta frequency bands in waking EEG.** EEG: Whole EEG segment; Cont-: removing 5% of contiguous EEG values from the middle of each segment; Rand-: removing 5% of EEG values at random; Avs-: Removing only 2.5 *SD* positive and negative LVDs from each segment (4.2% of points total on average). For waking EEG (14 subjects, 8 × 1 min EEG each), spectral power via FFT was computed in each segment, and averaged across all subjects and all segments. For each frequency band, the mean sum of spectral power is displayed. Alpha: 8–13 Hz; Beta: 16–31 Hz; Gamma: 35–45 Hz. *p < 0.01 for repeated measures ANOVA vs. whole EEG segment.

In order to assess whether >2.5 *SD* EEG LVDs contribute to the spectral structure of EEG, we removed all EEG values containing 2.5 *SD* positive and negative LVDs from the 14 subjects’ waking consciousness tracings, or about 4.2% of voltage readings per segment on average (Figure 5). For comparison, we included both complete waking EEG tracings, and waking EEG tracings where 5% of the voltage values were removed randomly from each segment, and waking EEG tracings where 5% of contiguous EEG data was excised from the middle of the segment (Figure 5). FFT was performed on these data, and spectral power in the alpha (8–13 Hz), beta (16–31 Hz), and gamma (35–45 Hz) frequency bands was summed for each of eight waking EEG segments from the 14 participants (Figure 5; Table 4). Compared to complete waking EEG, random deletion of either 5% of the voltage values or a segment of 5% of contiguous values from the middle of the EEG tracing had minimal effect on the resulting mean spectral power in the frequency bands, whereas removing both positive and negative 2.5 *SD* LVDs significantly reduced mean spectral power in the alpha and beta bands (Figure 5; Table 4). Therefore, 2.5 *SD* LVDs characterized by DSI contribute substantially to various spectral features in EEG.

**Table 4:** **Removal of 2.5 *SD* LVDs reduces alpha and beta spectral power**.

	Power	*F*_(1, 209)_	*p*-value^1^
**Alpha**
EEG	0.36 (0.48)
vs.
Avs-	0.23 (0.34)	10.04	0.0018**
Cont-	0.36 (0.49)	0	1
Rand-	0.36 (0.48)	0	1
**Beta**
EEG	0.071 (0.083)
vs.
Avs-	0.06 (0.065)	12.3	0.0006**
Cont-	0.071 (0.083)	0.002	0.96
Rand-	0.071 (0.083)	0	1
**Gamma**
EEG	0.018 (0.034)
vs.
Avs-	0.016 (0.028)	1.13	0.29
Cont-	0.018 (0.034)	0	1
Rand-	0.018 (0.034)	0.004	0.95

^1^Listed p values uncorrected for multiple comparisons. EEG, entire EEG segment. Avs-, positive and negative 2.5 SD. LVDs removed. Cont-, 5% contiguous segment of EEG removed from the middle of segment. Rand-, 5% of values removed randomly from segment. **p < 0.01 after Bonferroni correction for multiple comparisons.

## Discussion

### EEG-Derived LVDs Exhibit DSI

It has been well-established that power-law dynamics of cortical neuronal avalanches differ little between *in vitro* and *in vivo* animal models and human MEG (i.e., Plenz and Thiagarajan, 2007; Ribeiro et al., 2010; Shriki et al., 2013). Given that the EEG is likely determined by time-dependent dynamical neuronal avalanches, we reasoned that such structure in the waking ACF could indicate the loss of some measure of scale invariance in the neuronal avalanche distribution. The central finding of our study is that DSI is characteristic of the PDFs of EEG-derived LVDs in both waking and sleep stage 2 consciousness (Figure 3). Therefore, there is likely to be widespread coordination of the sizes of cortical neuronal avalanches in cortical brain activity, indicating a characteristic “lacunarity” in the power-law distribution (Sornette, 1998, 2006).

### DSI is more Prevalent in Waking than Sleep Stage 2

Our study showed that the total number of significant Lomb-Scargle peaks of positive and negative 2.5 *SD* LVDs derived from 1 min EEG segments was greater in waking than in sleep stage 2, and the total significant Lomb-Scargle power was greater in negative LVDs for waking than sleep stage 2 (Figure 3; Table 2). These results strongly suggest that improved EEG datasets would demonstrate a stronger difference in many measures of DSI between waking and sleep 2. Given that sleep stage 2 EEG had less overall variance than waking, fewer segments of sleep stage 2 consciousness gave >50 LVDs per segment than in waking (Table 1); for the purposes of this study, these segments were eliminated from further analysis, but this may have led to overestimating the level of DSI in sleep stage 2 compared to waking. While this is not proof, it is a strong inference that DSI is more prominent in waking, compared to sleep stage 2.

### Limitations

We utilized a small, publicly-available database with limited demographic and clinical information, so certainly these results should be repeated on larger, more complete datasets. While we demonstrated the presence of DSI in EEG-derived LVDs, we cannot demonstrate at this time that the degree of DSI can completely account for regular structures in seen in EEG FFT or ACF analysis (Figures 1, 1, 5). Our definition of EEG-derived LVDs (derived from a single lead) lacks a spatial component, which may limit the applicability of these results to other studies of neuronal avalanche dynamics. While we have observed a difference in DSI derived from EEG LVDs between waking and sleep stage 2, these differences may be characteristic only of sleep, and not characteristic of other unconscious brain states (i.e., anesthesia, coma). More definitive evidence that DSI of EEG-derived LVDs is attenuated in states of reduced conscious awareness will have to await analysis with better datasets.

### DSI of EEG LVDs: a New Clue in the Study of Consciousness?

The study of consciousness has long fascinated scientists, and much has been learned about the phenomenology (Boly et al., 2013) and neurobiology (Edelman and Tonioni, 2000; Baars et al., 2013) of conscious mental states. Additionally, much recent work has gone into the role of LRTC in states of consciousness and brain pathology (Li et al., 2008; Nikulin et al., 2012; Blythe et al., 2014). However, a neural dynamical understanding of consciousness has been elusive. We believe that the finding that DSI is more prominent in conscious mental states could be an important observation that will lead a paradigm shift in the understanding of the neurobiology of consciousness, as it would be able to link the behavioral manifestations of consciousness with a specific statistical physical pattern of neuronal activation via EEG. These provocative findings, while preliminary, do raise the hypothesis that the degree of DSI in EEG LVDs is intimately linked to cortical functioning in consciousness. In this hypothesis, conscious brain states would be characterized by increased DSI as compared to unconscious brain states. However, this excitement should be tempered by the preliminary nature of these findings. Certainly many more studies in both *in vitro* and *in vivo* systems are needed to confirm and expand upon these observations in order to have a strong indication that LVD DSI is indeed important for the understanding of consciousness. Nonetheless, we find these early results compelling, and worthy of future investigation.

## Author Contributions

TZ came up with the initial ideas for this work. TZ and MM jointly performed all the experiments listed, discussed findings and interpretations, and wrote the manuscript.

## Conflict of Interest Statement

The authors declare that the research was conducted in the absence of any commercial or financial relationships that could be construed as a potential conflict of interest.

## References

[B1] Arciniegas D. B. (2011). Clinical electrophysiologic assessments and mild traumatic brain injury: state-of-the-science and implications for clinical practice. Int. J. Psychophysiol. 82, 41–52. 10.1016/j.ijpsycho.2011.03.004 21419178

[B2] Baars B. J. Franklin S. Ramsoy T. Z. (2013). Global workspace dynamics: cortical “binding and propagation” enables conscious contents. Front. Psychol. 4:200. 10.3389/fpsyg.2013.00200 23974723 PMC3664777

[B3] Blythe D. A. Haufe S. Müller K. R. Nikulin V. V. (2014). The effect of linear mixing in the EEG on Hurst exponent estimation. Neuroimage 99, 377–387. 10.1016/j.neuroimage.2014.05.041 24862080

[B4] Boly M. Seth A. K. Wilke M. Ingmundson P. Baars B. Laureys S.. (2013). Consciousness in humans and non-human animals: recent advances and future directions. Front. Psychol. 4:625. 10.3389/fpsyg.2013.00625 24198791 PMC3814086

[B5] Coeurjolly J.-F. (2001). Simulation and identification of the fractional Brownian motion: a bibliographic and comparative study. J. Stat. Softw. 5, 1–53. 10.18637/jss.v005.i07

[B6] de Arcangelis L. Herrmann H. J. (2012). Activity-dependent neuronal model on complex networks. Front. Physiol. 3:62. 10.3389/fphys.2012.00062 22470347 PMC3314197

[B7] Edelman G. M. Tonioni G. (2000). A Universe of Consciousness: How Matter Becomes Imagination. New York: Basic Books.

[B8] Gireesh E. D. Plenz D. (2008). Neuronal avalanches organize as nested theta- and beta/gamma-oscillations during development of cortical layer 2/3. Proc. Natl. Acad. Sci. U S A 105, 7576–7581. 10.1073/pnas.0800537105 18499802 PMC2396689

[B9] Goldberger A. L. Amaral L. A. N. Glass L. Hausdorff J. M. Ivanov P. C. Mark R. G.. (2000). PhysioBank, PhysioToolkit and PhysioNet: components of a new research resource for complex physiologic signals. Circulation 101, E215–E220. 10.1161/01.cir.101.23.e215 10851218

[B10] Ichimaru Y. Moody G. B. (1999). Development of the polysomnographic database on CD-TOM. Psychiatry Clin. Neurosci. 53, 175–177. 10.1046/j.1440-1819.1999.00527.x 10459681

[B11] Kantelhardt J. W. Zschiegner S. A. Koscielny-Bunde E. Havlin S. Bunde A. Stanley H. E. (2002). Multifractal detrended fluctuation analysis of nonstationary time series. Physica A Stat. Mech. Appl. 316, 87–114. 10.1016/s0378-4371(02)01383-3

[B12] Kaplan A. Ya. Fingelkurts A. A. Fingelkurts A. A. Borisov S. V. Darkhovsky B. S. (2005). Nonstationary nature of the brain activity as revealed by EEG/MEG: methodological, practical and conceptual challenges. Signal Process. 85, 2190–2212. 10.1016/j.sigpro.2005.07.010

[B13] Kitzbichler M. G. Smith M. L. Christensen S. R. Bullmore E. (2009). Broadband criticality of human brain network synchronization. PLoS Comput. Biol. 5:e1000314. 10.1371/journal.pcbi.1000314 19300473 PMC2647739

[B14] Klaus A. Yu S. Plenz D. (2011). Statistical analyses support power law distributions found in neuronal avalanches. PLoS One 6:e19779. 10.1371/journal.pone.0019779 21720544 PMC3102672

[B15] Li Y. Qiu J. Yang Z. Johns E. J. Zhang T. (2008). Long-range correlation of renal sympathetic nerve activity in both conscious and anesthetized rats. J. Neurosci. Methods 172, 131–136. 10.1016/j.jneumeth.2008.04.015 18511128

[B16] Muzy J. F. Bacry E. Arneodo A. (1993). Multifractal formalism for fractal signals: the structure-function approach versus the wavelet-transform modulus-maxima method. Phys. Rev. E Stat. Phys. Plasmas Fluids Relat. Interdiscip. Topics 47, 875–884. 10.1103/physreve.47.875 9960082

[B17] Nikulin V. V. Jönsson E. G. Brismar T. (2012). Attenuation of long-range temporal correlations in the amplitude dynamics of alpha and beta neuronal oscillations in patients with schizophrenia. Neuroimage 61, 162–169. 10.1016/j.neuroimage.2012.03.008 22430497

[B18] Nunez P. L. (2010). Brain, Mind, and the Structure of Reality. New York: Oxford University Press.

[B19] Nunez P. L. Srinivasan R. (2006). Electric Fields of the Brain: The Neurophysics of EEG. New York: Oxford University Press.

[B20] Palva J. M. Zhigalov A. Hirvonen J. Korhonen O. Linkenkaer-Hansen K. Palva S. (2013). Neuronal long-range temporal correlations and avalanche dynamics are correlated with behavioral scaling laws. Proc. Natl. Acad. Sci. U S A 110, 3585–3590. 10.1073/pnas.1216855110 23401536 PMC3587255

[B21] Plenz D. Thiagarajan T. C. (2007). The organizing principles of neuronal avalanches: cell assemblies in the cortex? Trends Neurosci. 30, 101–110. 10.1016/j.tins.2007.01.005 17275102

[B22] Poil S. S. Hardstone R. Mansvelder H. D. Linkenkaer-Hansen K. (2012). Critical-state dynamics of avalanches and oscillations jointly emerge from balanced excitation/inhibition in neuronal networks. J. Neurosci. 32, 9817–9823. 10.1523/JNEUROSCI.5990-11.2012 22815496 PMC3553543

[B23] Press W. H. Teukolsky S. A. Vetterling W. T. Flannery B. P. (2007). Numerical Recipes: The Art of Scientific Computing. New York: Cambridge University Press.

[B24] Priesemann V. Valderrama M. Wibral M. Le Van Quyen M. (2013). Neuronal avalanches differ from wakefulness to deep sleep-evidence from intracranial depth recordings in humans. PLoS Comput. Biol. 9:e1002985. 10.1371/journal.pcbi.1002985 23555220 PMC3605058

[B25] RCoreTeam (2012). R: A Language and Environment for Statistical Computing. Vienna: R Foundation for Statistical Computing.

[B26] Ribeiro T. L. Copelli M. Caixeta F. Belchior H. Chialvo D. R. Nicolelis M. A.. (2010). Spike avalanches exhibit universal dynamics across the sleep-wake cycle. PLoS One 5:e14129. 10.1371/journal.pone.0014129 21152422 PMC2994706

[B27] Schiff N. D. Nauvel T. Victor J. D. (2014). Large-scale brain dynamics in disorders of consciousness. Curr. Opin. Neurobiol. 25C, 7–14. 10.1016/j.conb.2013.10.007 24709594 PMC3980494

[B28] Schwilden H. (2006). Concepts of EEG processing: from power spectrum to bispectrum, fractals, entropies and all that. Best Pract. Res. Clin. Anaesthesiol. 20, 31–48. 10.1016/j.bpa.2005.09.001 16634412

[B29] Shew W. L. Plenz D. (2013). The functional benefits of criticality in the cortex. Neuroscientist 19, 88–100. 10.1177/1073858412445487 22627091

[B30] Shriki O. Alstott J. Carver F. Holroyd T. Henson R. N. Smith M. L.. (2013). Neuronal avalanches in the resting MEG of the human brain. J. Neurosci. 33, 7079–7090. 10.1523/JNEUROSCI.4286-12.2013 23595765 PMC3665287

[B31] Sornette D. (1998). Discrete scale invariance and complex dimensions. Phys. Rep. 297, 239–270. 10.1016/s0370-1573(97)00076-8

[B32] Sornette D. (2006). Critical Phenomena in Natural Sciences Chaos, Fractals Selforganization and Disorder: Concepts and Tools. Berlin: Springer-Verlag.

[B33] Weisstein E. W. (2014). “Fourier transform.” From MathWorld–A Wolfram Web Resource. Available online at: http://mathworld.wolfram.com/FourierTransform.html (accessed November 21, 2015).

[B34] Yu S. Yang H. Shriki O. Plenz D. (2013). Universal organization of resting brain activity at the thermodynamic critical point. Front. Syst. Neurosci. 7:42. 10.3389/fnsys.2013.00042 23986660 PMC3749752

[B35] Zhou W.-X. Sornette D. (2002). Generalized q-analysis of log-periodicity: applications to critical ruptures. Phys. Rev. E Stat. Nonlin. Soft Matter Phys. 66:046111. 10.1103/physreve.66.046111 12443263

[B36] Zhou W. X. Sornette D. Hill R. A. Dunbar R. I. (2005). Discrete hierarchical organization of social group sizes. Proc. Biol. Sci. 272, 439–444. 10.1098/rspb.2004.2970 15734699 PMC1634986

[B37] Zorick T. Mandelkern M. A. (2013). Multifractal detrended fluctuation analysis of human EEG: preliminary investigation and comparison with the wavelet transform modulus maxima technique. PLoS One 8:e68360. 10.1371/journal.pone.0068360 23844189 PMC3700954

